# Involvement of autotaxin in the pathophysiology of elevated intraocular pressure in Posner-Schlossman syndrome

**DOI:** 10.1038/s41598-020-63284-1

**Published:** 2020-04-14

**Authors:** Nozomi Igarashi, Megumi Honjo, Reiko Yamagishi, Makoto Kurano, Yutaka Yatomi, Koji Igarashi, Toshikatsu Kaburaki, Makoto Aihara

**Affiliations:** 1 Department of Ophthalmology, Graduate School of Medicine, The University of Tokyo, Tokyo, Japan; 2 Department of Clinical Laboratory Medicine, Graduate School of Medicine, The University of Tokyo, Tokyo, Japan; 3 Department of Clinical Laboratory, The University of Tokyo Hospital, Tokyo, Japan; 4 Bioscience Division, Reagent & Development Management, TOSOH Corporation, Kanagawa, Japan

**Keywords:** Glaucoma, Uveal diseases, Biochemistry, Predictive markers, Prognostic markers

## Abstract

To examine whether autotaxin (ATX) in the aqueous humor causes elevated intraocular pressure (IOP) in patients with Posner-Schlossman syndrome (PSS). ATX and transforming growth factor beta (TGF-β) in the aqueous humor were quantified in PSS patients. The expression of ATX and TGF-β in cytomegalovirus (CMV)-infected-human trabecular meshwork (hTM) cells was examined. Biological changes in hTM cells and monkey Schlemm’s canal endothelial (SCE) cells cultured in the conditioned medium of CMV-infected hTM cells were analyzed. The expression of ATX and TGF-β1 was upregulated in the aqueous humor of CMV-positive PSS patients, and the level of ATX in the aqueous humor was positively correlated with IOP. CMV infection upregulated ATX and TGF-β1 in hTM cells. The conditioned medium induced fibrotic changes in hTM cells and reduced SCE permeability, which was attenuated by an ATX inhibitor, a lysophosphatidic acid receptor antagonist, and a Rho kinase inhibitor. ATX in the aqueous humor induced by CMV infection may trigger elevated IOP. Modulating ATX activity may be a novel treatment modality for PSS.

## Introduction

Glaucoma is the second leading cause of blindness globally. It is characterized by a marked increase in intraocular pressure (IOP) that causes damage to the optic nerve^1–3^. Impairment of aqueous humor (AH) drainage through the trabecular outflow pathway is the primary cause of elevated IOP in glaucoma patients. Elevated IOP is the most important risk factor in all glaucoma subtypes, and lowering IOP delays loss of vision in glaucoma patients^2–6^.

Elevated IOP is an uncommon but serious complication in uveitic glaucoma^7^, especially in cases where the uveitis is long standing or recurrent. IOP may remain raised for long periods of time and can be particularly refractive in certain types of uveitic glaucoma^8^ such as Posner-Schlossman syndrome (PSS)^9–11^.

PSS causes a higher rate of elevated IOP compared to other types of uveitis, and the rate of glaucomatous optic neuropathy is reported to be as high as 45%^12,13^. PSS is characterized by unilateral anterior uveitis (AU), causing recurrent attacks of marked IOP elevation^13^. Young males are most frequently affected in PSS, and the total duration of the disease is highly related to the progression of glaucoma^14^. Hirose *et al*., have previously reported that HLA (human leukocyte antigen) -Bw54 and haplotype HLA-Bw54-Cw1 showed significant association with PSS in a Japanese population, so HLA may have also be an important factor in glaucomatocyclitic crisis^15^. Recently, in clinical practice, PCR (polymerase chain reaction) testing of AH has enabled the detection of viral DNA (deoxyribonucleic acid) and enabled diagnosis of the cause in cases of AU that were previously labelled idiopathic. In addition, several studies have reported the identification of herpes virus in AH samples from patients with hypertensive refractive AU^16–19^. Among herpes viruses, it has been reported that cytomegalovirus (CMV) has a particularly strong association with PSS, with up to 52.2% of PSS patients testing positive for CMV^18,20^. It has also been suggested that a higher CMV copy number is a substantial risk factor for refractive IOP elevation^21^.

Topical medication including corticosteroids, anti-glaucoma agents, and ganciclovir can control inflammation and the elevation of IOP in most PSS cases; however, some patients are resistant to treatment and must undergo surgical procedures^19,22^. CMV-positive PSS patients are more likely to have refractive symptoms and undergo operations including filtration surgery compared to CMV-negative PSS patients^19^.

In CMV-positive PSS patients, increased IOP is caused by dysfunction in the trabecular meshwork (TM) or Schlemm’s canal endothelium (SCE) cells and the upregulation of several cytokines in the AH. However, the mechanism is still not well understood. For example, transforming growth factor-β (TGF-β) 2 is increased in the AH of patients with primary open-angle glaucoma (POAG) and is considered a mediator to regulate fibrotic response in the TM and increase outflow resistance in the conventional outflow pathway^23^. It has been reported that CMV infection induces secretion of TGF-β1 in cultured cells^24,25^, and TGF-β 2 was increased in the AH of patients with PSS compared with controls^26^. However, the latter study found no difference between CMV-positive and CMV-negative PSS regarding the cytokine profile in the AH, including TGF-β1, TGF-β2, IL (interleukin) -8, and IL-10^26^. Thus, TGF-β cannot be the cause of increased IOP elevation in CMV-positive PSS patients.

To improve the prognosis of CMV-positive PSS patients, it is necessary to first understand the mechanisms underlying elevated IOP. Recently, we reported that autotaxin (ATX), a generating enzyme for lysophosphatidic acid (LPA), has a significant impact on IOP in different glaucoma subtypes and fibrotic changes in the TM, especially in secondary glaucoma^27,28^. Thus, in the present study, we explored whether the level of aqueous ATX is altered in CMV-positive PSS patients, and whether ATX or TGF-β are related to elevated IOP. We also examined the effect of a conditioned medium derived from CMV-infected TM cells on fibrotic changes in the TM and resistance in conventional aqueous outflow.

## Results

### Demographic data of the study population

Table 1 lists the demographic data of the study population including CMV-positive and CMV-negative PSS patients. Of the 19 eyes from 19 CMV-positive PSS patients in this study, 9 eyes were diagnosed as having SOAG (secondary open angle glaucoma), i.e., PSS (CMV+/SOAG+); and 10 eyes were CMV-positive without SOAG, PSS (CMV+/SOAG−). Seven eyes with PSS only (CMV-/SOAG−) were included as a control (Table 1).

**Table 1:** Demographic characteristics of the study population.

Variables	CMV−/SOAG−	CMV+/SOAG−	CMV+/SOAG+	*P-*value
Patients (n)	7	10	9	
Number of eyes (n)	7	10	9	
Gender (male:female)	6:1	10:0	6:3	NS*
**Age (years)**				
Mean ± SD	53.7 ± 19.6	64.3 ± 9.9	60.9 ± 13.4	
[range]	22–75	43–80	39–86	NS*
**IOP (mmHg)**				
Mean ±SD	22.1 ± 8.0	18.0 ± 5.9	21.3 ± 6.5	
[range]	14–38	8–31	12–34	NS**
**Number of glaucoma eye drops**				
Mean ± SD	0.3 ± 0.5	0.7 ± 0.7	2.3 ± 1.0	
[range]	0–1	0–2	0–4	†<.0001, ††<.0001
Phakia vs IOL (n)	7:0	8:2	4:5	NS*
**Aqueous ATX level (μg/L)**				
Mean ±SD	531.2 ± 121.2	520.5 ± 54.7	707.4 ± 137.0	†<.005, ††<.005
[range]	384.9–676.2	405.9–673.9	410.1–979.4	

CMV, cytomegalovirus; SOAG, secondary open angle glaucoma; IOP, intraocular pressure; IOL, intraocular lens.

*Fisher’s exact test; **Kruskall-Wallis.

^†^Statistically significant difference between CMV(+)SOAG(+) and CMV(−)SOAG(−) (Steel-Dwass test).

^††^Statistically significant difference between CMV(+)SOAG(+) and CMV(+)SOAG(−) (Steel-Dwass test).

The gender ratios (male/female) for each group were 6:1, 10:0, and 6:3 for PSS (CMV-/SOAG−), PSS (CMV+/SOAG−), and PSS (CMV+/SOAG+), respectively. Although there was no significant difference in IOP among the three groups, the number of glaucoma eye drops used was significantly different: 0.3 ± 0.5 for PSS (CMV-/SOAG−), 0.7 ± 0.7 for PSS (CMV+/SOAG−), and 2.3 ± 1.0 for PSS (CMV+/SOAG+) (Table 1).

**Figure 1:**
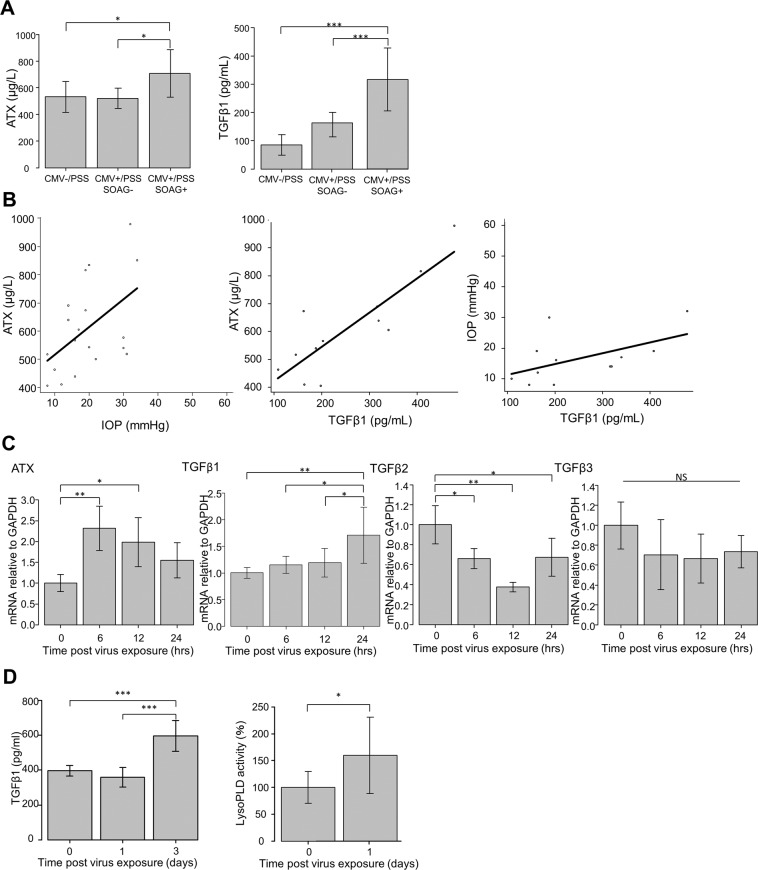
Relationships between ATX or TGF-β1 level in aqueous humor of PSS patients and quantification of ATX and TGF-β in CMV-infected hTM cells and conditioned medium. (**A**,**B**) Relationships between ATX or TGF-β1 level in aqueous humor of PSS patients. (**A**) Left) The ATX level was significantly higher in the PSS (CMV+/SOAG+) group than in the PSS (CMV−/SOAG−) or PSS (CMV+/SOAG−) groups (*P* < 0.05). Right) The TGF-β1 level was significantly higher in the PSS (CMV+/SOAG+) group than in the PSS (CMV-/SOAG−) or PSS (CMV+/SOAG−) groups (*P* < 0.001). (**B**) Left) IOP and ATX were significantly correlated in CMV-positive PSS patients (Spearman’s rank correlation coefficient = 0.523, *P* = 0.0215). Middle) ATX and TGF-β1 were significantly correlated in CMV-positive PSS (Spearman’s rank correlation coefficient = 0.692, *P* = 0.0159). Right) TGF-β1 and IOP were not significantly correlated (Spearman’s rank correlation coefficient = 0.552, *P* = 0.0629). (**C**) qPCR quantification of ATX and TGF-β1, 2 and 3 in CMV-infected hTM cells. The relative mRNA expression of ATX (**A**) and TGF-β1 (**B**) was significantly higher in CMV-infected cells compared to the control. TGF-β2 showed decreased expression post-CMV infection (**C**) and TGF-β3 showed no significant changes (**D**). RT-qPCR with GAPDH primers was performed to serve as an internal control for input DNA. Data are the averages of four independent DNA samples from the infected cells. Values are the mean ± standard error. ^*^P < 0.05, ^**^P < 0.01. (**D**) TGF-β1 and LysoPLD activity in the conditioned medium. Both TGF-β1 (**A**) and LysoPLD activity (**B**) in the conditioned medium were elevated post-infection. TGF-β1 was significantly higher at 3 dpi, and LysoPLD activity was increased at 1 dpi.

First, we compared the levels of ATX and TGF-β1, 2, and 3 between eyes without any complications (control) and PSS (CMV+/SOAG+) (Supplemental Table 1 and Fig. 1). We found significant differences in ATX and TGF-β1 between the control group and the PSS (CMV+/SOAG+) group (Supplemental Fig. 1A,B), but there were no differences in TGF-β2 and 3 levels (Supplemental Fig. 1C,D). Therefore, we speculated that ATX or TGF-β1 but not TGF-β2 might playing a major role in the elevation of IOP in PSS (CMV+/SOAG+), so we explored ATX and TGF-β1 levels in more detail in the PSS (CMV-/SOAG−), PSS (CMV+/SOAG−), and PSS (CMV+/SOAG+) groups. We excluded patients with CMV-negative PSS without SOAG, as we were unable to fully document the presence or absence of viral infections other than CMV.

The level of ATX was significantly higher in the PSS (CMV+/SOAG+) group than in the PSS (CMV-/SOAG−) and PSS (CMV+/SOAG−) groups (*P* < 0.05 for each group; Table 1, Fig. 1A). We also compared the aqueous TGF-β1 level between groups, and found it was significantly higher in the PSS (CMV+/SOAG+) group (*P* < 0.05 for each group; Fig. 1A).

IOP was positively correlated with the level of aqueous ATX (Fig. 1B). The aqueous levels of TGF-β1 were positively correlated with the levels of aqueous ATX in the CMV + groups (Fig. 1B). However, there were no significant correlations between IOP and TGF-β1 (Fig. 1B) or TGF-β2 and 3 (data not shown) in either of the CMV + groups.

These results suggest that ATX or TGF-β1 in the AH of PSS patients may increase IOP by affecting the outflow pathway through the TM and Schlemm’s canal. Thus, we hypothesized that aqueous ATX or TGF-β1 may be produced in the TM in response to CMV infection, which may induce fibrotic changes or increase resistance in the aqueous outflow pathway through the accumulation of extracellular matrix (ECM) or changes in cytoskeleton and cell-cell contacts.

### Confirmation of CMV infection

First, CMV-infected hTM cells were prepared and stained with anti-cytomegalovirus antibody, clone 8B1.2, to check the expression of IE (immediate early) antigen. IE antigen was detected 6 h after exposure to cell-free CMV medium (Supplemental Fig. 2).

### CMV-induced mRNA expression of ATX and TGF-**β**1, 2, and 3

We analyzed the mRNA expression levels of ATX and TGF-β1, 2, and 3 in CMV-infected hTM cells using qRT-PCR (Fig. 1C). Basal levels of ATX and TGF-β1, 2, and 3 were detectable in CMV-infected hTM cells (Fig. 1C). Compared with the control, CMV infection significantly increased the expression of ATX at 6 h (*P* < 0.01) and 12 h (*P* < 0.05) post-virus exposure (Fig. 1C). TGF-β1 expression was also significantly higher at 24 h post-infection than in the control (*P* < 0.01), and in the 6- and 12-h post-infection groups (*P* < 0.05) (Fig. 1C). CMV infection significantly decreased the expression of TGF-β2 at 6, 12, and 24 h (*P* < 0.05) compared to the control (Fig. 1C). There was no difference in relative mRNA expression of TGF-β3 after virus exposure (Fig. 1C).

### Secreted TGF-**β**1 and activity of ATX in the conditioned medium

The levels of secreted TGF-β1 in the conditioned medium were measured using ELISA (Enzyme-Linked Immuno Sorbent Assay). The levels of TGF-β1 were significantly higher in the CMV-infected conditioned medium at 3 days post infection (dpi) (Fig. 1D), but not at 1 dpi. LysoPLD (lysophospholipase D) activity was significantly higher in the conditioned medium than in the control at 1 dpi (Fig. 1D).

### The expression of ATX and TGF-β1 in CMV-infected hTM assessed by immunocytochemistry and western blotting

**Figure 2:**
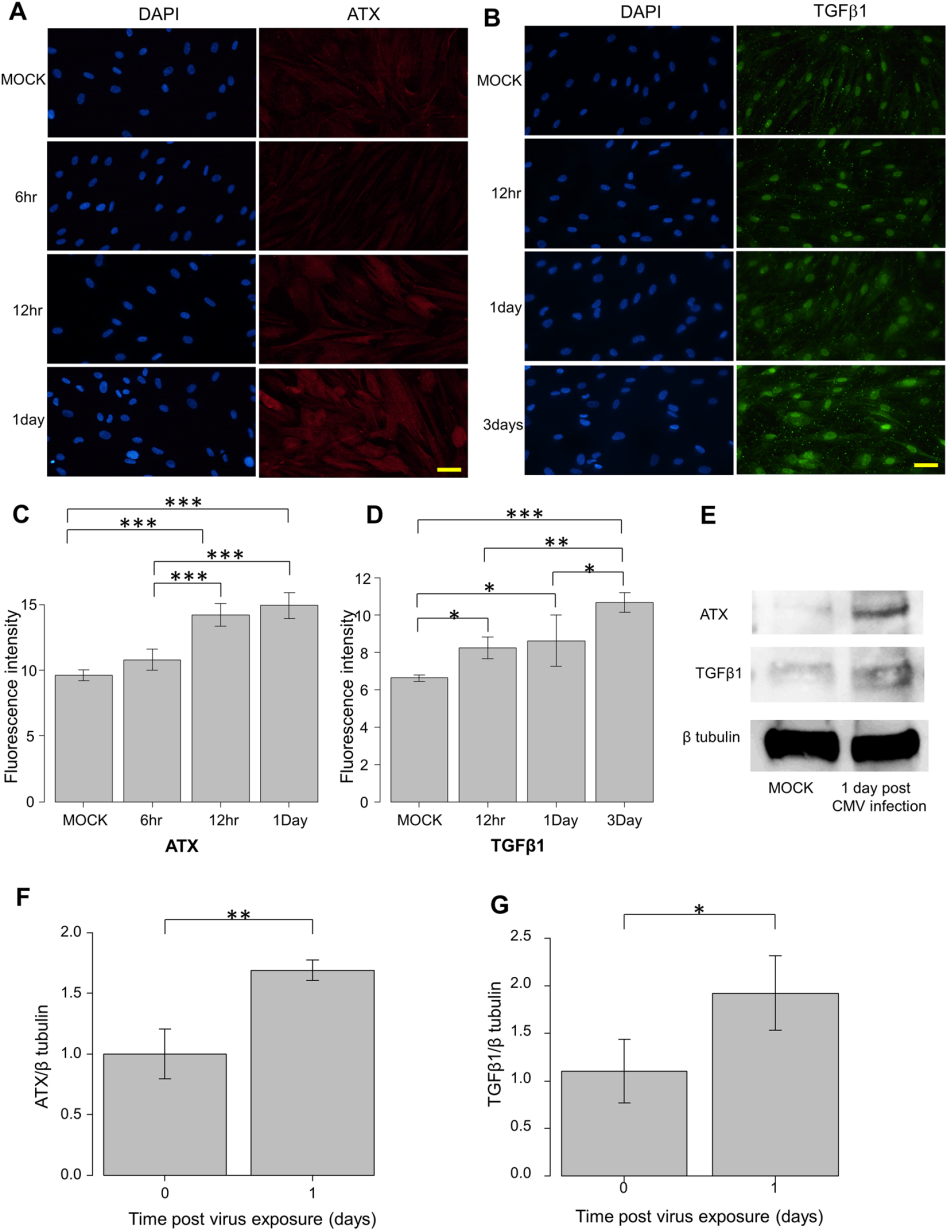
Immunocytochemistry and western blotting of ATX and TGF-β1 in CMV-infected hTMs. (**A**,**B**) Immunocytochemistry of ATX and TGF-β1 in CMV-infected hTMs. The left panels show cells that were stained with DAPI. The right panels show cells stained for ATX (**A**) or TGF-β1 (**B**). The right panels show the merged image. For ATX images (**A**), the expression in the control is shown in the first row, followed by 6, 12, and 24 h post-infection. For the TGF-β1 images (**B**), the control is shown in the first row, followed by 12, 24, and 72 h post-infection. The expression of both ATX and TGF-β1 increased post-infection, and the expression increased over time. Bar, 200 μm. (**C**,**D**) Quantitative results based on immunocytochemistry. Four images of each experiments were taken and the fluorescence intensities were quantified. Data were presented as the mean ± standard deviation. ^*^P < 0.05, ^**^P < 0.01, ^***^P < 0.001. (**E**–**G**) Western blotting of ATX and TGF-β1 in CMV-infected hTMs. The representative bands for ATX and TGF-β1 are shown in (**C**), and the relative expression of ATX (**D**) and TGF-β1 (**E**) compared to the loading control (β-tubulin) are shown (n = 3). The grouping of gels/blots cropped from different parts of the same gel. ^*^P < 0.05, ^**^P < 0.01.

Immunocytochemistry and western blotting were used to assess the protein expression of ATX and TGF-β1 post-CMV infection. Figure 2A–D shows the immunocytochemistry results. The expression of ATX and TGF-β1 was significantly upregulated at 1 dpi for ATX (Fig. 2A) and at 1–3 dpi for TGF-β1 (Fig. 2B). And also the quantified intensities showed that both ATX and TGF-β1 were upregulated with CMV infection (Fig. 2C,D). Figure 2E shows the data from western blotting (n = 3) confirming the upregulation of ATX and TGF-β1 protein expression (Fig. 2E), and the quantified analysis shows that the expression of ATX and TGF-β1 was significantly upregulated at 1 dpi (Fig. 2F,G).

### Fibrotic changes in hTM cells induced by conditioned medium

Next, fibrotic changes in hTM cells were assessed by immunohistochemistry. The role of ATX in the conditioned medium was confirmed using an ATX inhibitor (HA130), as well as an LPA receptor antagonist (Ki16425). In addition, Rho kinase (ROCK) inhibitors (Y27632, K115) were also used because the Rho-ROCK pathway is downstream of LPA and TGF-β signaling. Downstream cascade activation of TGF-β was also inhibited by SB431542, which is a selective inhibitor of activin-like kinase (ALK) receptors.

**Figure 3:**
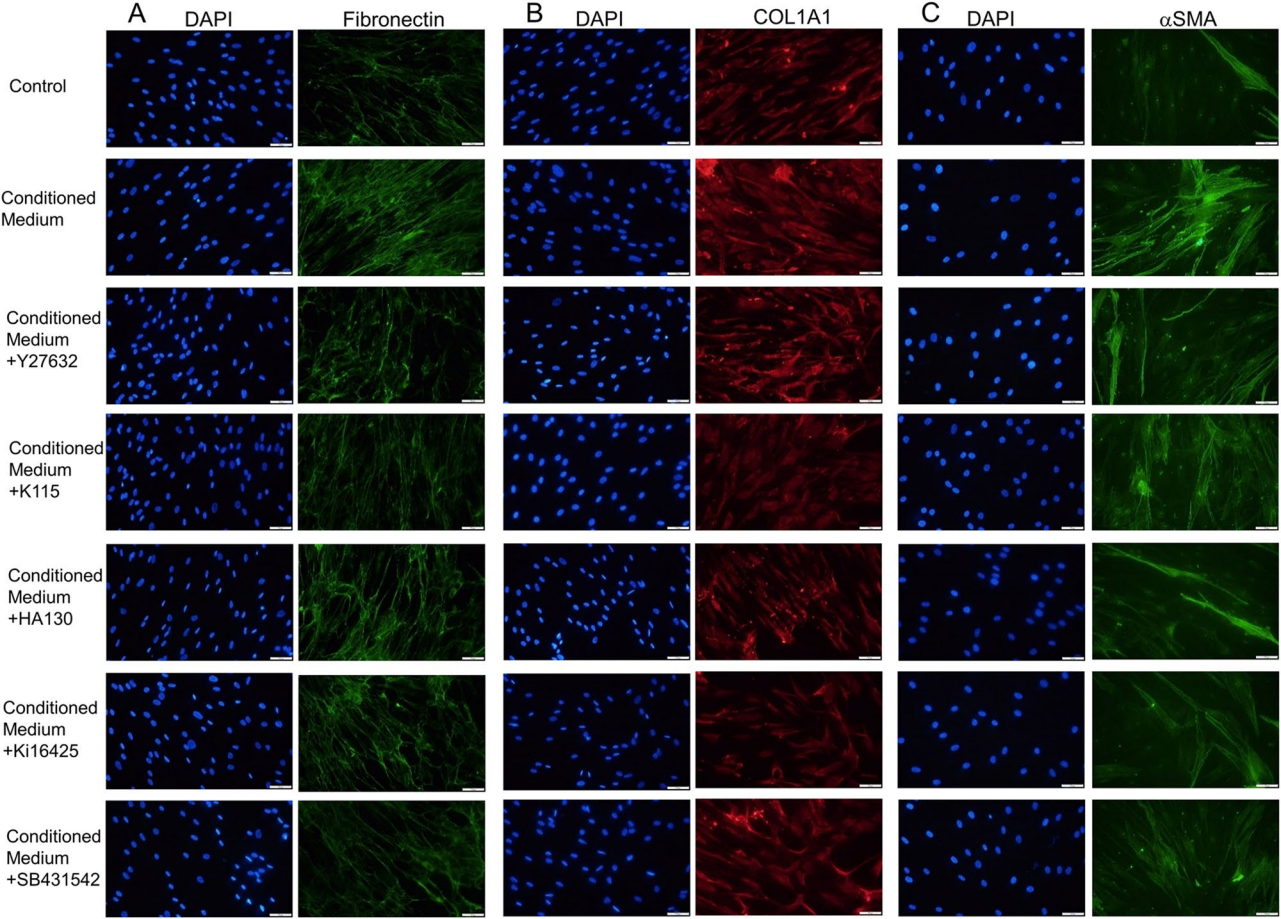
Immunocytochemistry of fibronectin, COL1A1, and αSMA in hTMs exposed to the conditioned medium. The left panels show cells that were stained with DAPI. The middle panels show cells stained for fibronectin, COL1A1, and αSMA. The right panels show the merged image. The expression of fibronectin, COL1A1, and αSMA increased post-infection, and were attenuated with each inhibitor or antagonist application. Bar, 200 μm.

**Figure 4:**
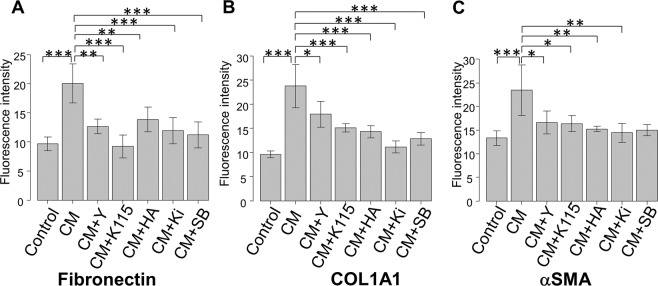
Quantitative results based on immunocytochemistry of fibronectin, COL1A1, and αSMA in hTMs exposed to the conditioned medium. Four images of each experiments were taken and the fluorescence intensities were quantified for fibronectin (**A**), COL1A1 (**B**) and αSMA (**C**). Data were presented as the mean ± standard deviation. ^*^P < 0.05, ^**^P < 0.01, ^***^P < 0.001.

Figure 3 shows that the expression of fibronectin, COL1A1, and αSMA were upregulated at 1 dpi in hTM. The conditioned medium induced a significant level of fibronectin (Fig. 3A), COL1A1 (Fig. 3B), and αSMA (alpha smooth muscle actin) (Fig. 3C) expression. This effect was attenuated by the ATX inhibitor (HA130), the LPA receptor antagonist (Ki16425), ROCK inhibitors (Y27632 and K115), and the ALK receptor inhibitor (SB431542). And also the quantified intensities showed the same tendency (Fig. 4A–C).

### Cell-cell contact and cell permeability in monkey SCE cells assessed by immunocytochemistry and FITC (Fluorescein isothiocyanate)-dextran flux

**Figure 5:**
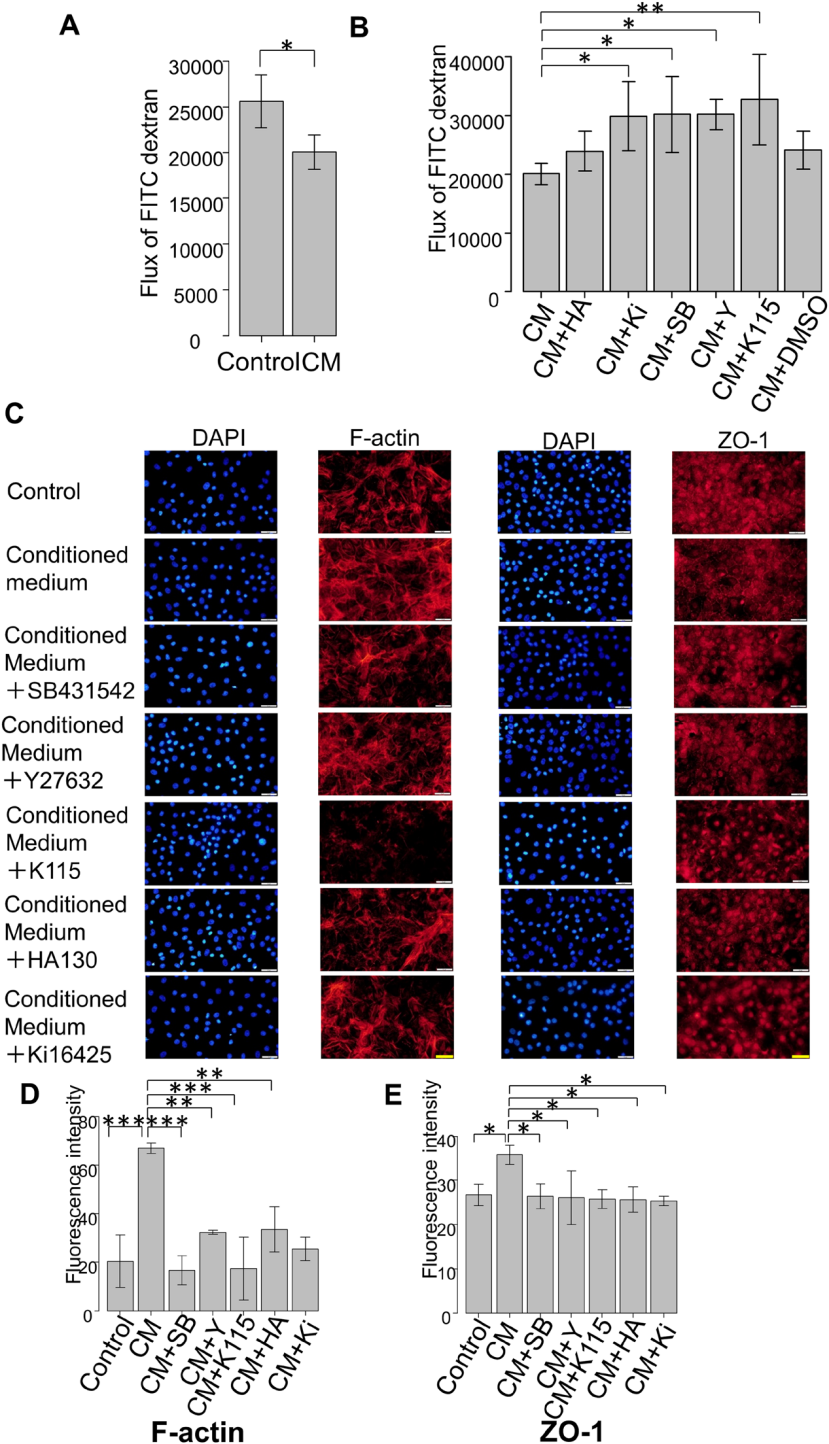
Measurement of monolayer cell permeability in SCE cells exposed to the conditioned medium with or without inhibitors and immunocytochemistry of F-actin in MSCs exposed to the conditioned medium. (**A**,**B**) Changes in the SCE cell monolayer permeability using 4 kDa FITC-dextran are shown. SCE cells were exposed to the conditioned medium, and concentrations were measured at 6 h post-exposure. Mean values from four separate filters are presented. (**A**) SCE cell monolayer permeability was significantly reduced after exposure. (**B**) SCE cell monolayer permeability increased in the presence of the inhibitors (Y27632, K115, Ki16425, and SB431542). *P < 0.05, ^**^P < 0.01. (**C**) Immunocytochemistry in MSCs exposed to the conditioned medium. The left panels show cells that were stained with DAPI. The middle panels show cells stained for F-actin and ZO-1. The control expression is shown in the first row, conditioned medium exposure in the second row, cells exposed to conditioned medium under SB431542 application in the third row, under Y27632 application in the fourth row, under K115 application in the fifth row, under HA130 application in the sixth row, and under Ki16425 application in the seventh row. F-actin and ZO-1 expression increased post-exposure, which was attenuated in the presence of the inhibitors/antagonists. Bar, 200 μm. (**D**,**E**) Quantitative results based on immunocytochemistry. Four images of each experiments were taken and the fluorescence intensities were quantified. Data were presented as the mean ± standard deviation. ^*^P < 0.05, ^**^P < 0.01, ^***^P < 0.001.

Next, in an attempt to elucidate the mechanism of fibrotic changes in the outflow pathway, we focused on the SCE layer connected to the TM, through which the aqueous humor flows into the Schlemn’s canal. We performed permeability assays using a flux of FITC-dextran in SCE cells. Treatment with the conditioned medium significantly increased the concentration of FITC-dextran in the apical side of SCE cells at 6 h post exposure (*P* < 0.05; Fig. 5A). Additionally, when each inhibitor and antagonist was applied in combination with the conditioned medium, the ROCK inhibitors (Y27632 and K115) significantly attenuated the changes induced by the conditioned medium (*P* < 0.05 and *P* < 0.01, respectively, Fig. 5B). This suggests that upregulation of the Rho-ROCK pathway is involved in the decreased permeability of SCE cells. In addition, the LPA receptor antagonist (Ki16425) and inhibitor of TGF-β receptor (SB431542) also attenuated the changes induced by the conditioned medium, although Ki16425 did not cause a significant change (*P* < 0.05, Fig. 5B).

The immunohistochemical analysis revealed that the expression of F-actin and ZO (zonula occludens)-1 was significantly higher in SCEs exposed to the conditioned medium compared to levels in the control group (Fig. 5C). As shown in Fig. 5C, treatment with the conditioned medium resulted in robust expression of F-actin in SCE cells, which was significantly suppressed by the ROCK inhibitors (Y27632 and K115), ATX inhibitor (HA), LPA antagonist (Ki16425), and SB431542. As for the expression of ZO-1 (Fig. 5C), upregulated expression of ZO-1 induced by the conditioned medium was significantly suppressed by the ATX, TGF-β, and ROCK inhibitors as well as the LPA antagonist, which supports the results of the SCE permeability experiment (Fig. 5B). And also the quantified intensities showed the same tendency (Fig. 5D,E).

## Discussion

In the present study, we found that AH samples from PSS (CMV+/SOAG+) patients showed significantly higher levels of ATX and TGF-β1 compared to control samples, and significantly higher ATX levels than compared to PSS (CMV+/SOAG−) samples. More importantly, the level of aqueous ATX was positively correlated with IOP, whereas no significant correlation between TGF-β1 and IOP was observed. Moreover, *in vitro* experiments revealed that CMV infection upregulated ATX and TGF-β1 and induced fibrotic changes in hTM cells, while also significantly reducing SCE permeability, which was attenuated by inhibitors of the ATX-LPA-ROCK pathway.

Several studies concerning changes in aqueous cytokine profiles in different clinical entities of uveitis, including infectious uveitis^29^, Bechet’s disease, Vogt-Koyanagi-Harada disease^30,31^, Fuchs heterochromic cyclitis, and other clinically idiopathic uveitis^32–37^ have reported that increased levels of IL-6, CCL (C-C motif ligand) 2, and CXC Chemokine Ligand (CXCL) 8 are the most commonly observed changes associated with ocular inflammatory disease in general, and similar changes in these cytokines were also reported in PSS patients^26^. However, we saw significantly higher levels of IL-6 and CXCL8 in CMV-negative PSS patients compared to CMV-positive PSS patients (data not shown). Although the existence of CMV DNA in the AH of PSS patients and the positive correlation between CMV and refractive IOP elevation have been reported by several groups, the effect of aqueous CMV on intraocular inflammation and IOP elevation remain unknown, which encouraged us to further investigate the effects of CMV-infection on the cytokine profile, regulation of fibrotic changes, and aqueous outflow resistance in the TM.

Our results showed a significant increase in the level of ATX in the AH of PSS (CMV+/SOAG+) patients compared to that of PSS (CMV-/SOAG−) patients (Fig. 1A). This suggests a relationship between CMV infection and ATX secretion in the AH. In addition, the level of ATX in PSS (CMV+/SOAG+) patients was significantly higher than that in PSS (CMV+/SOAG−) patients (Fig. 1A). Moreover, there was a correlation between ATX and increased IOP in CMV-positive PSS patients (Fig. 1B). In a previous study, we reported that ATX was upregulated in the AH as well as in the outflow pathway in SOAG patients and that it may be involved in the inflammatory changes in TM that cause elevated IOP^28^. Several previous studies have also shown that cytoskeletal changes induced by ECM deposition and fibrosis of HTM cells impair AH outflow through the TM, leading to IOP elevation. These changes appear to involve the Rho/ROCK pathway, which has been implicated in the control of the contractile and biomechanical properties of the TM and Schlemm’s canal^38,39^. We saw a similar correlation between increased ATX and elevated IOP in our previous study. It is possible that ATX, which is an enzyme which catalyzes the conversion of LPC (lysophosphatidylcholine) to LPA, may be a potential target to treat TM or Schlemm’s canal dysfunction.

We found that the levels of TGF-β1 in the AH of PSS (CMV+/SOAG+) patients were significantly higher than those in PSS (CMV-/SOAG−) or PSS (CMV+/SOAG−) patients (Fig. 1A). There were no significant differences in TGF-β2 or 3 (Supplemental Fig. 1) levels. Previous studies also found that CMV induced the secretion of TGF-β1 in TM cells^40^. However, unlike ATX, we saw no significant correlation between TGF-β1 and IOP in CMV-positive PSS patients. It is well known that TGF-β2 isoforms induce TM and ciliary muscle contraction, accelerating ECM deposition to regulate outflow resistance in the conventional POAG pathway^23,41,42^. Although TGF-β1 has been implicated to play a possibly important role in the regulation of outflow resistance^43,44^, and higher levels of the TGF-β1 isoform occur as seen most commonly in pseudoexfoliation glaucoma^41^, it has been speculated that the high IOP itself might induce the expression of activated TGF-β1 in trabecular meshwork cells^45^. Compared to TGF-β2, the role of TGF-β1 in IOP regulation in open angle glaucoma is not yet well understood, so further studies would be needed to clarify the effects of TGF-β1 in OAG^23^. Therefore, we conducted further *in vitro* investigation concerning the levels and effects of TGF-β1.

To confirm the relationship between CMV infection, ATX, and IOP elevation, we conducted *in vitro* studies to determine the expression of ATX and TGF-β in CMV-infected hTM cells, and also explored whether CMV infection causes fibrotic changes in hTM cells and increased permeability in SCE cells. First, we confirmed CMV infection in hTM cells by ICC 6 h after infection (Supplemental Fig. 2). qPCR, immunocytochemistry, and western blotting showed that CMV infection in hTM cells significantly increased ATX and TGF-β1 expression (Fig. 1C). As shown in Fig. 1C, we found that gene expression of ATX precedes the gene expression of TGF-β1. LPA activates the autocrine TGF-β1-Smad signaling pathway, which induces expression of TGF-β1, and also has protective effects such as anti-fibrogenesis in corneal fibroblast cells^46–48^. Because ATX is an upstream enzyme for LPA production, it is possible that higher expression of ATX in CMV-infected hTMs may lead to increased LPA, and in turn, increased TGF-β1. In contrast, we observed that TGF-β2 expression was suppressed after CMV infection (Fig. 1C). Further studies are needed to elucidate this relationship.

To clarify the effects of cytokine changes induced by CMV infection, we applied the conditioned medium to TM cells and SCEs and looked for fibrotic changes and an increase in cell permeability. We first measured the TGF-β1 level and LysoPLD activity in the conditioned medium and found that both were significantly higher than in the control (Fig. 1D). We also found that elevated LysoPLD activity preceded the elevation in TGF-β1.

The conditioned medium induced significant fibrotic changes in TM cells, suggesting that upregulated ATX and possibly TGF-β1 can cause paracrine effects. These fibrotic changes were significantly attenuated by the ATX inhibitor, LPA receptor antagonist, ROCK inhibitors, and the TGF-β receptor inhibitor (Figs. 3A–C, 4A–C). Therefore, the regulation of these signaling pathways may be a target for a novel therapeutic modality.

In addition to the changes in TM cells, SCE cell permeability decreased immediately after exposure to the conditioned medium (Fig. 5A). Compared to rapid changes of outflow facility mediated by TGFβ2^38^, it has been reported that the effect of TGFβ1 to cause IOP elevation follows after the effect of TGFβ2 to elevate IOP^49^. This decreased permeability was attenuated by the LPA receptor antagonist, ROCK inhibitors, and TGF-β receptor inhibitor (Fig. 5B). This suggests that the upregulation of Rho-ROCK induced by the ATX-LPA pathway was responsible for the decreased permeability of SCE cells. As confirmed in Fig. 5C–E, SCEs exposed to the conditioned medium showed higher expression of ZO-1 and F-actin. Our present findings support the hypotheses that dysfunction in TM and SCE cells induced by CMV infection causes rapid changes in aqueous outflow resistance in the Schlemm’s canal, causing extremely elevated IOP (higher than 30 mmHg). We can assume that upregulation of ATX along with LPA combined with prolonged TGF-β1 elevation contribute to resistance at the Schlemm’s canal, causing refractive, prolonged IOP elevation in CMV-positive PSS patients. In addition, ROCK inhibitors have the greatest potential to weaken cell-cell contact and improving outflow resistance at the level of Schlemm’s canal. Therefore, they are an attractive target for novel treatment of refractive IOP elevation in CMV-positive PSS patients.

Our study has several limitations. First, we collected the AH during the peak of IOP elevation, and had no baseline for comparison. We also lacked a baseline for measuring the CMV copy number or activity of the CMV infection. Further investigations will need to include prospective studies. Second, although ATX and TGF-β1 in the anterior chamber were positively correlated, we couldn’t fully describe this phenomenon. Further studies to assess this correlation are needed. Third, AH samples from CMV-positive or -negative patients are limited, thus, more CMV+/SOAG + AH characteristics should be documented in future research. Fourth, this is a retrospective and *in vitro* study, thus further experiments using animal models to explore whether CMV infection in the anterior chamber induces ATX and TGF-β1 upregulation or IOP elevation are needed in the future. Fifth, we could not investigate the relationship between CMV infection and possible transcriptional factors for ATX. Several transcriptional factors have been implicated for ATX up-regulation in other cells, including c-Jun and STAT3 (signal transducer and activator of transcription 3)^50–52^. We would like to further explore molecular basis for the CMV induced autotaxin upregulation in the trabecular meshwork cells in the future study. Finally, it has been shown that the ATX inhibitor used in the present study can reduce ATX activity quickly with low cytotoxicity *in vitro* and in animal model, but we will need more specific selective inhibitor when we aim the clinical use. In the future, an alternative ATX inhibitor may show more significant effects on CMV-induced pathogenesis.

In the present study, we found that ATX is upregulated in aqueous humor and hTMs with CMV infection and may be triggering the fibrotic changes in hTM cells and higher resistance at Schlemm’s canal. Modulating ATX and its downstream cascade could be used as a novel modality for patients with CMV positive PSS to improve the acute and recurrent attack.

## Methods

### Patients and aqueous humor samples

AH samples were obtained from patients who underwent cataract surgery or patients with clinical signs of PSS who underwent PCR testing of aqueous humor samples from March 2014 to July 2018 at the University of Tokyo Hospital. This prospective observational study was approved by the Institutional Review Board of the University of Tokyo and was registered with the University Hospital Medical Information Network Clinical Trials Registry of Japan (ID: UMIN000027137). All of the procedures conformed to the Declaration of Helsinki. Written informed consent was obtained from each patient.

Inflammation was analyzed using the criteria set by the Standardization of Uveitis Nomenclature (SUN) working group for scoring the anatomical location, onset, duration, course, and activity of the disease^53^. The diagnosis criteria for PSS are as follows: recurrent elevated IOP higher than 21 mmHg, mild anterior chamber inflammation (anterior chamber cells: occasional to 1+ SUN grade) with fine to medium keratic precipitates and unilateral eye involvement. Patients suffering from known systemic, genetic or infectious causes for the inflammation were excluded, because they may have other types of ocular diseases. And, patients with a previous history of intraocular surgery other than small incision cataract surgery without complications were excluded. All patients were negative for tuberculosis, sarcoidosis, syphilis, herpes simplex virus, varicella-zoster virus, rubella virus, and toxoplasmosis genomic DNA in the AH samples. The IOP was determined using a Goldmann tonometer at outpatient clinic (from 9 am to 5 pm) at patients’ visit when the aqueous humor was collected. When both eyes of a patient met the inclusion criteria, only the eye treated first was included in the analyses. For all patients, the anterior eye segment and optic disc were examined by glaucoma or uveitis specialists using a slit-lamp biomicroscope and dilated fundoscopy to diagnose glaucoma.

### Aqueous humor collection

AH samples were collected as described previously^27^. Briefly, in eyes with PSS, AH was collected in the outpatient clinic before the commencement of treatment. Under topical anesthesia, approximately 70–100 μL AH was obtained using a 30-gauge syringe, collected in a PROTEOSAVE SS 1.5 mL Slimtube (Sumitomo Bakelite, Tokyo, Japan), registered, and stored at −80 °C until processing. For cataract controls, AH was collected at the beginning of cataract surgery after paracentesis was performed.

### Measurement of ATX, ATX isoforms, and TGF-β1, 2 and 3 in the AH

The level of ATX in the AH was determined using a two-site immunoenzymatic assay with an ATX assay reagent equipped with a Tosoh AIA system (Tosoh, Tokyo, Japan) as described previously^27,28,54,55^. The TGF-β levels in the AH were measured using the Bio-Plex Pro TGF-β Assay (Bio-Rad, CA, USA) following the manufacturer’s protocol.

### Culture of human trabecular meshwork cells

hTM cells were purchased from ScienCell Research Laboratories (San Diego, CA, USA) and cultured in Dulbecco’s modified Eagle’s medium (DMEM) containing 2% fetal bovine serum (FBS) and Antibiotic Antimycotic Solution (100×) (Sigma-Aldrich, St. Louis, MO, USA) at 37 °C in 5% CO_2_. We used three biological replicates, and cells were verified and characterized by immunofluorescence with antibodies specific to α-SMA and fibronectin. Cells from passages 3–5 were used in the experiments.

### Culture of monkey Schlemm’s canal endothelial (SCE) cells

The SCE explants dissected from the eyes of 6-12-month-old cynomolgus monkeys were obtained from a commercial laboratory (Shin Nippon Biomedical Laboratories, Kagoshima, Japan). We used modified dissection methods based on previous work by Alvarado *et al*.^56–58^ Primary SCE cells were cultured in DMEM containing 10% FBS and Antibiotic Antimycotic Solution (100×) (Sigma-Aldrich) at 37 °C in 5% CO_2_, and SCE cells from passages 4–6 were used in all experiments.

### Virus infection and collection of conditioned medium

A telomerase-immortalized human fibroblast cell line, hTERT-BJ1 (Invitrogen, Carlsbad, CA, USA), was grown in DMEM:199 (4:1) supplemented with 10% FBS and infected with human CMV Towne strain AD169 at a multiplicity of infection (MOI) of 0.4. After culturing for 5 days, culture supernatants were harvested, followed by centrifugation for 10 min at 3,000 rpm at room temperature to prepare the cell-free CMV medium. The harvested medium was used after one freeze/thaw cycle. After reaching confluence, hTM cells were incubated with the cell-free CMV medium for 2 h at 37 °C in 5% CO_2_ with a MOI of 1. After 2 h, the medium was removed, and the infected cells were washed twice with phosphate-buffered saline (PBS) and fresh growth medium was added.

One to three days post-infection (dpi), the infected hTM growth medium was removed, centrifuged, frozen at −70 °C, and used as the conditioned medium for subsequent experiments.

### Immunocytochemistry

Immunocytochemistry was performed as previously described^55^. The primary antibodies were Anti-Cytomegalovirus Antibody, clone 8B1.2 (1:2,000; Merck Millipore, Billerica, MA, USA), Anti-ENPP2 (ectonucleotide pyrophosphatase/phosphodiesterase family member 2) antibody [5H3] (1:1,000; Abcam, Cambridge, MA, USA), Anti-TGF-β1 antibody (1:100; Sigma-Aldrich), anti-αSMA (1:500; Sigma-Aldrich) and anti-fibronectin [IST-9] (1:400; Abcam). Alexa Fluor 488 and 594 secondary antibodies (1:1,000) were purchased from Thermo Fisher Scientific (Waltham, MA, USA). To assess the characteristic changes in hTM and SCE cells after exposure to the conditioned medium, we performed immunocytochemistry using rhodamine phalloidin (7:1,000, Thermo Fisher Scientific) and ZO-1 (1:100; Abcam), followed by Alexa Fluor 488 and 594 secondary antibodies (1:1000; Thermo Fisher Scientific). We further explored whether the changes induced by the conditioned medium could be suppressed by ROCK inhibitors Y27632 (Merck, Kenilworth, NJ, USA) and K115 (KOWA, Nagoya, Japan), as well as Ki16425 (Merck) and SB431542 (Fujifilm, Osaka, JAPAN).

### Quantitative PCR

The cells were lysed using TRI REAGENT (Molecular Research Center, Inc., Cincinnati, OH, USA), and mRNA was isolated using chloroform and isopropyl alcohol as previously described^55^. The mRNA was treated with a PrimeScript RT Reagent Kit (Takara Bio, Shiga, Japan) to synthesize cDNA. mRNA was quantified using quantitative PCR (qPCR) with SYBR Premix Ex Taq II (Tli RNaseH Plus) (Takara Bio) and the Thermal Cycler Dice Real Time System II (Takara Bio) using the ΔΔCt method. For qPCR, primer sequences were taken from previously published sequences, and the primers were purchased from Hokkaido System Science (Hokkaido, Japan).

The sequences of the PCR primers were shown in Supplemental Table 2. The data were normalized relative to GAPDH.

### Western blotting

After 1 dpi, cells were collected in RIPA Buffer (Thermo Fisher Scientific) containing protease inhibitors (Roche Diagnostics, Basel, Switzerland), sonicated, and centrifuged. The following protein concentration measurement and SDS-PAGE was performed as previously described^55^. Protein bands were transferred to PVDF membranes (Bio-Rad Laboratories) and the membranes were immersed in Tris-buffered saline with Tween 20 (TBST) containing primary antibody. After washing, the membranes were immersed in TBST containing secondary antibody and reacted with ECL substrate (Thermo Fisher Scientific). Protein bands were detected by ImageQuant LAS 4000 mini (GE Healthcare, Chicago, IL, USA). The primary antibodies were anti-ENPP2 (1:1000; Abcam) and anti-β-tubulin (1:1000; Wako Pure Chemical Industries, Ltd., Osaka, Japan), and HRP-conjugated secondary antibody (1:2000; Thermo Fisher Scientific). The bands were quantified using ImageJ software (ver. 1.49, NIH, Bethesda, MD, USA).

### Measurement of TGF-β1 and LysoPLD activity in the conditioned medium

The level of secreted TGF-β1 in conditioned medium was measured using a Human TGF-β1 Quantikine ELISA Kit (R&D Systems, Inc., Minneapolis, MN, USA). The collected medium was activated prior to assay, and the ELISA protocol performed according to the manufacturer’s instructions.

ATX activity in the cultured medium was measured as lysoPLD activity, as previously described^59^. Briefly, lysoPLD activity was assessed by measuring choline liberation from the substrate LPC. The reactions were performed in 100 μL aliquots; 20 μL samples were incubated with 2 mM 1-myristoyl (14:0)-LPC in the presence of 100 mM Tris-HCl (pH 9.0) for 3 h at 37 °C. Liberated choline was detected using an enzymatic photometric method with choline oxidase, horseradish peroxidase, and a TOOS reagent as a hydrogen donor. The choline concentration was estimated with an absorption spectrometer.

### Measurement of monolayer cell permeability and immunocytochemistry in the SCE

To measure the monolayer cell permeability, SCE cells were grown on polycarbonate membrane inserts (0.4 μm pore size and 12 mm diameter; Corning Transwell; Sigma-Aldrich) on 12-well culture plates (BD Falcon, Franklin Lakes, NJ, USA) in DMEM supplemented with 10% FBS at 37 °C in 5% CO_2_ until confluent as previously described^60^. The volume of the applied medium was 0.5 mL on the apical side (inside of the membrane inserts) and 1.5 mL on the basal side (outside of the membrane inserts). Two weeks after seeding, SCE-cell monolayers were exposed to the conditioned medium, and a 4 kDa FITC-dextran dye (Sigma-Aldrich) was simultaneously applied at 50 μM to the basal compartment of the wells. The medium was collected from the apical side for fluorescence measurements at 3, 6, and 24 h after adding the dye, and the same volume of fresh culture medium was replaced. The concentration of FITC-dextran in the collected medium was measured using a multimode plate reader (Multi Microplate Reader, MTP-800AFC; Corona Electric, Ibaragi, Japan), with an excitation wavelength of 490 nm and an emission wavelength of 530 nm. Fluorescence intensity of the normal medium was measured as the background concentration in each experiment, and each experiment was repeated at least three times.

### Statistical analysis

Data were statistically analyzed using the EZR program (Saitama Medical Center, Hidaka, Japan)^61^. The results were expressed as the mean ± standard deviation (SD). The *t*-test and chi-square or Fisher’s exact test were used for comparing two variables, and the Steel-Dwass test was used for multiple variables. Differences in the data among the groups were analyzed by one-way analysis of variance and Tukey’s *post hoc* test. A value of *P* < 0.05 was considered statistically significant.

## Supplementary information

Supplementary Information.
