# Supplementary material for: Involvement of autotaxin in the pathophysiology of elevated intraocular pressure in Posner-Schlossman syndrome
Source: Sci Rep. 2020 Apr 14;10:6265. doi: 10.1038/s41598-020-63284-1 (PMC7156668; doi:10.1038/s41598-020-63284-1)

Involvement of autotaxin in the pathophysiology of elevated intraocular pressure in Posner-Schlossman syndrome

1. Nozomi Igarashi
Email: kindenauthor@yahoo.co.jp
Institution: The University of Tokyo
Department: Department of Ophthalmology
Address: 7-3-1 Hongo, Bunkyo-ku, Tokyo, Japan
Zip code: 113-8655
Work phone: 81-3-3815-5411

2. Megumi Honjo
Email: honjomegumi@gmail.com
Institution: The University of Tokyo
Department: Department of Ophthalmology
Address: 7-3-1 Hongo, Bunkyo-ku, Tokyo, Japan
Zip code: 113-8655
Work phone: 81-3-3815-5411

3. Reiko Yamagishi
Email: yamagishi015@gmail.com
Institution: The University of Tokyo
Department: Department of Ophthalmology
Address: 7-3-1 Hongo, Bunkyo-ku, Tokyo, Japan
Zip code: 113-8655
Work phone: 81-3-3815-5411

4. Makoto Kurano
Email: kurano-tky@umin.ac.jp
Institution: The University of Tokyo
Department: Department of Clinical Laboratory
Address: 7-3-1 Hongo, Bunkyo-ku, Tokyo, Japan
Zip code: 113-8655
Work phone: 81-3-3815-5411

5. Yutaka Yatomi
Email: yatoyuta-tky@umin.ac.jp
Institution: The University of Tokyo
Department: Department of Clinical Laboratory

Supplemental Figure 1

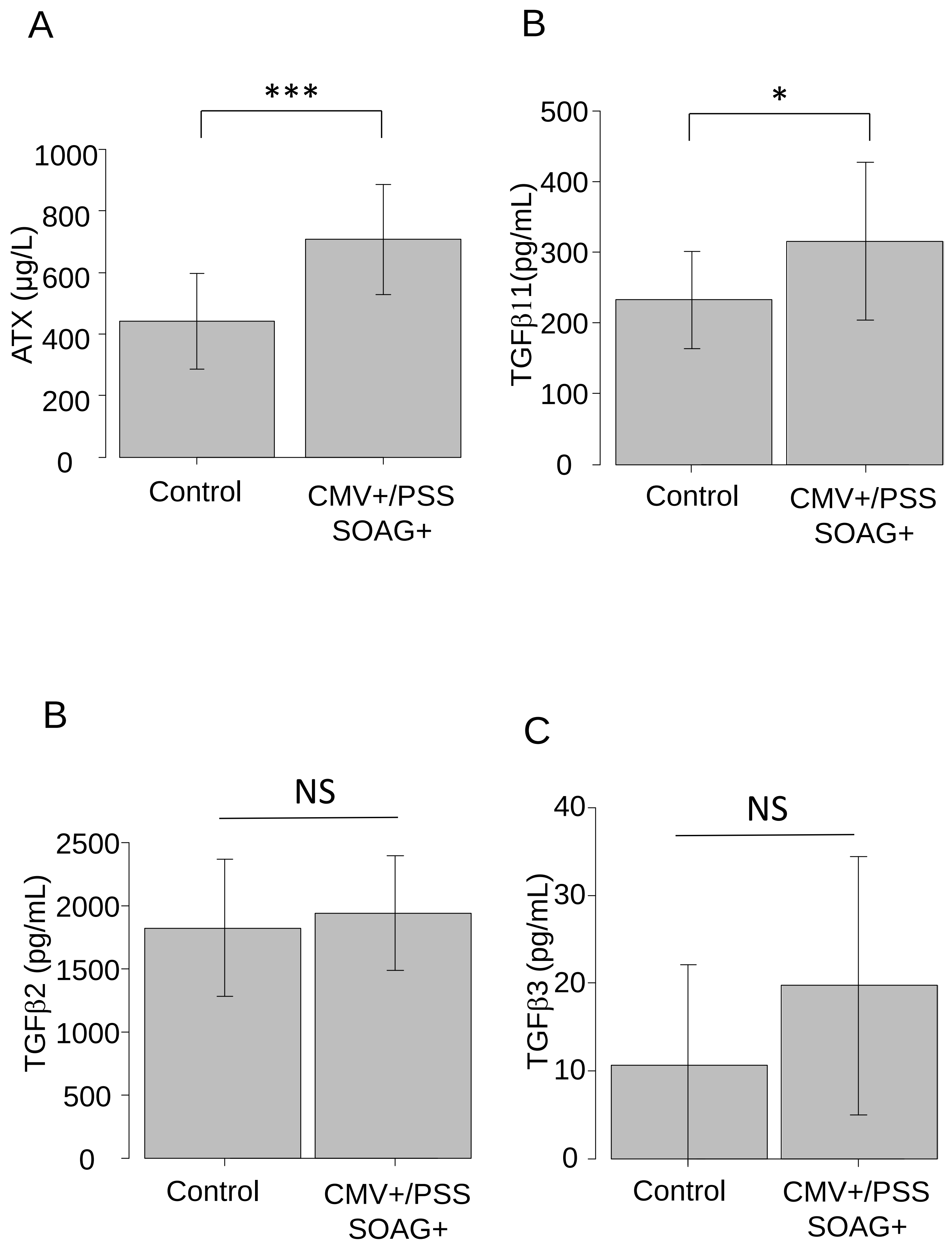

Supplemental Figure 2

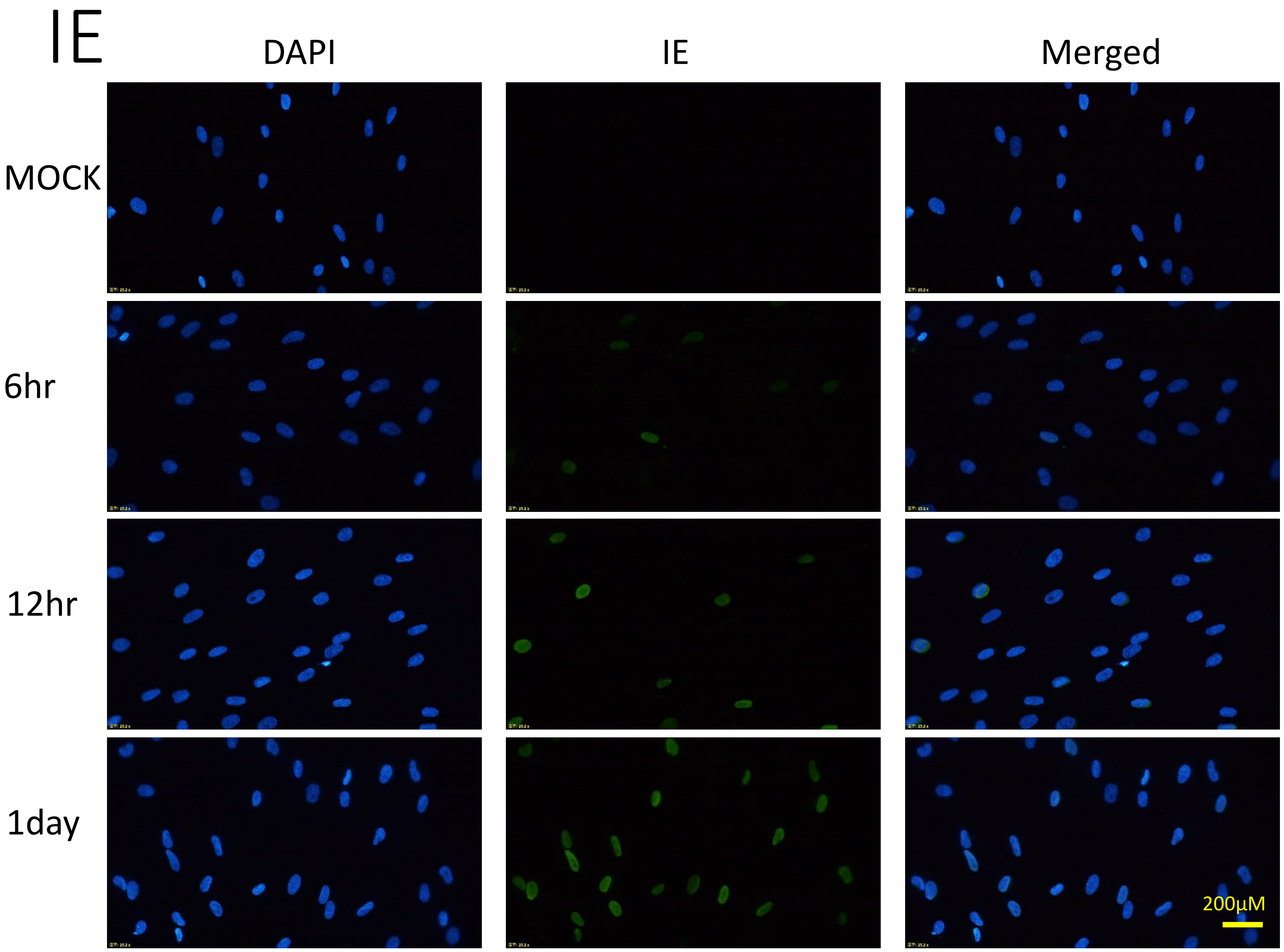

Supplemental Table 1. Demographic characteristics of the CMV+SOAG+ eyes and eyes without any ocular complications

Variables	Eyes without any complications	CMV+/SOAG	*P*-value
Patients (n)	17	9	
Number of eyes (n)	17	9	
Gender (male:female)	5:12	6:3	NS*
Age (years)			
Mean ± SD	73.5±3.8	60.9±13.4	
[range]	58-87	39-86	NS**
IOP (mmHg)			
Mean ± SD	13.5±0.7	21.3 ± 6.5	
[range]	12-16	12-34	NS**
Phakia vs IOL (n)	17:0	4:5	NS**
Aqueous ATX level (μg/L)			
Mean ± SD	441.2±79.5	707.4 ±137.0	†<.005,
[range]	196.7-717.3	410.1-979.4	

CMV, cytomegalovirus; SOAG, secondary open angle glaucoma; IOP, intraocular pressure; IOL, intraocular lens

*Fisher's exact test; ** Mann-Whitney U test

† statistically significant difference between CMV(+)SOAG(+) and Control groups (Steel-Dwass test)

SupplementalTable 2. The sequences of the PCR primers

PCR primers	
GAPDH	Forward: 5′-GAGTCAACGGATTTGGTCGT-3′
	Reverse: 5′-TTGATTTTGGAGGGATCTCG-3′
TGF-β1	Forward: 5′-CCCAGCATCTGCAAAGCTC-3′
	Reverse: 5′-GTCAATGTACAGCTGCCGCA-3′;
TGF-β2	Forward: 5′-TGCCGCCCTTCTTCCCCTC-3′
	Reverse: 5′-GGAGCACAAGCTGCCCACTGA-3′
TGF-β3	Forward: 5′-GGTTTTCCGCTTCAATGTGT-3′
	Reverse: 5′-TATAGCGCTGTTTGGCAATG-3′
ATX	Forward: 5′-ACAACGAGGAGAGCTGCAAT-3′
	Reverse: 5′-AGAAGTCCAGGCTGGTGAGA-3′.

Images used for the Western Blotting in Figure 2

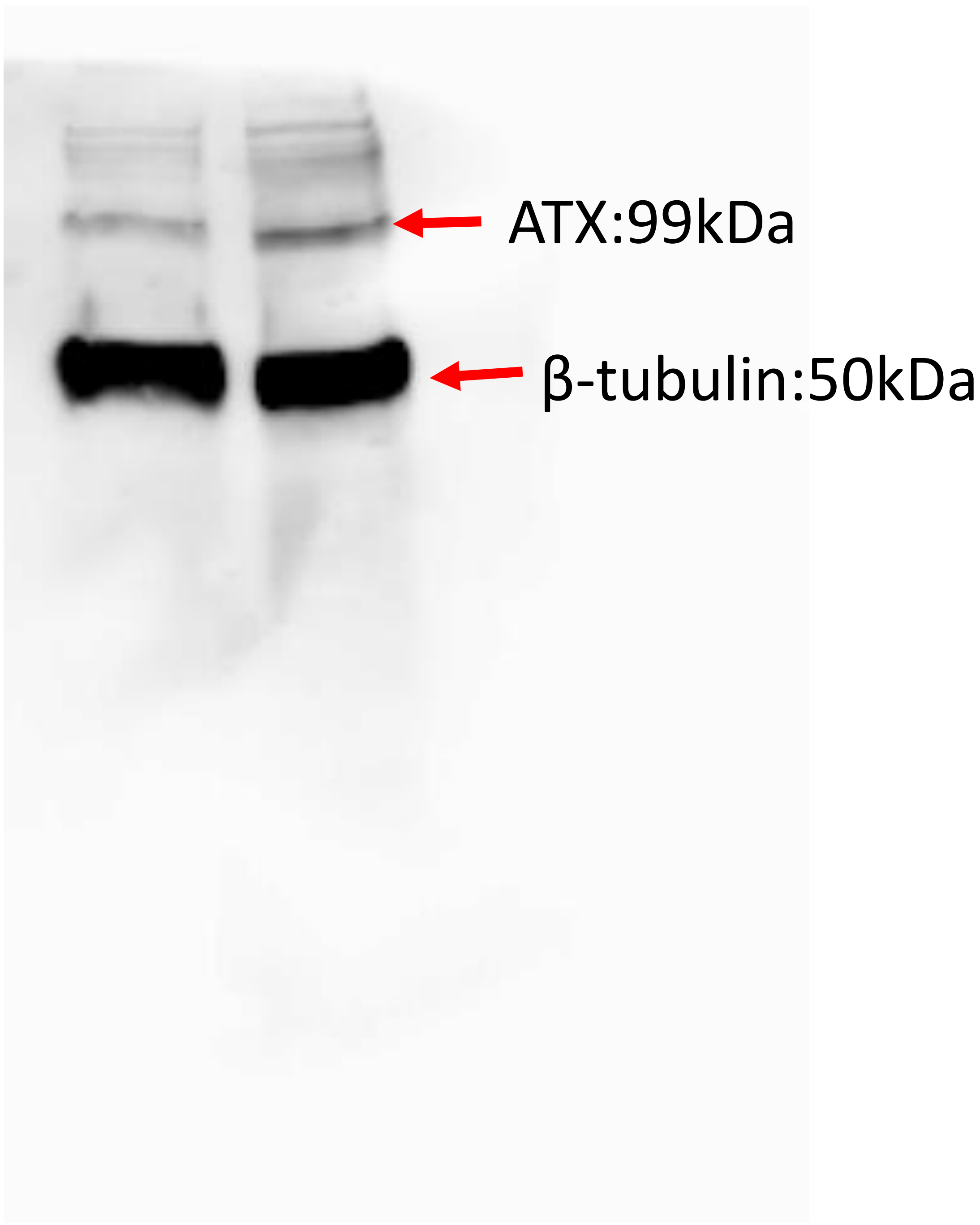

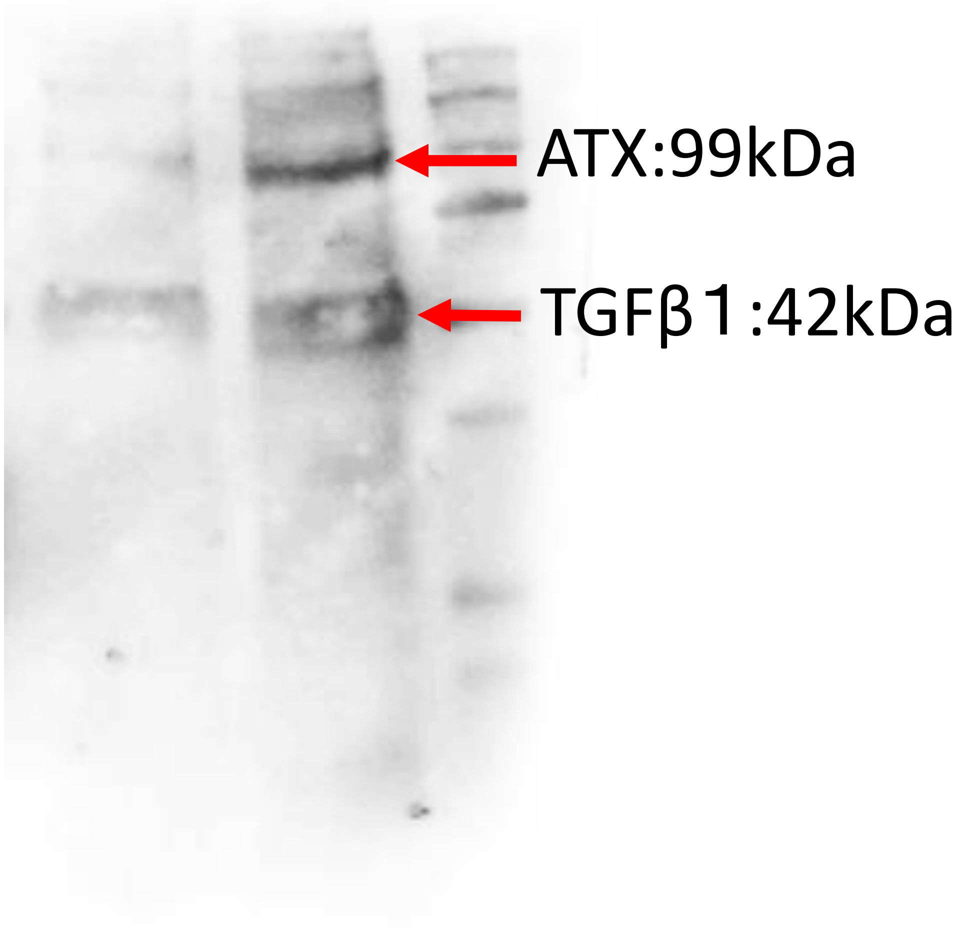

Figure shows the western blotting analysis of
protein harvested with or without CMV infection,
and those protein when loaded with recombinant ATX,
pictures taken under ATX antibody used in this manuscript

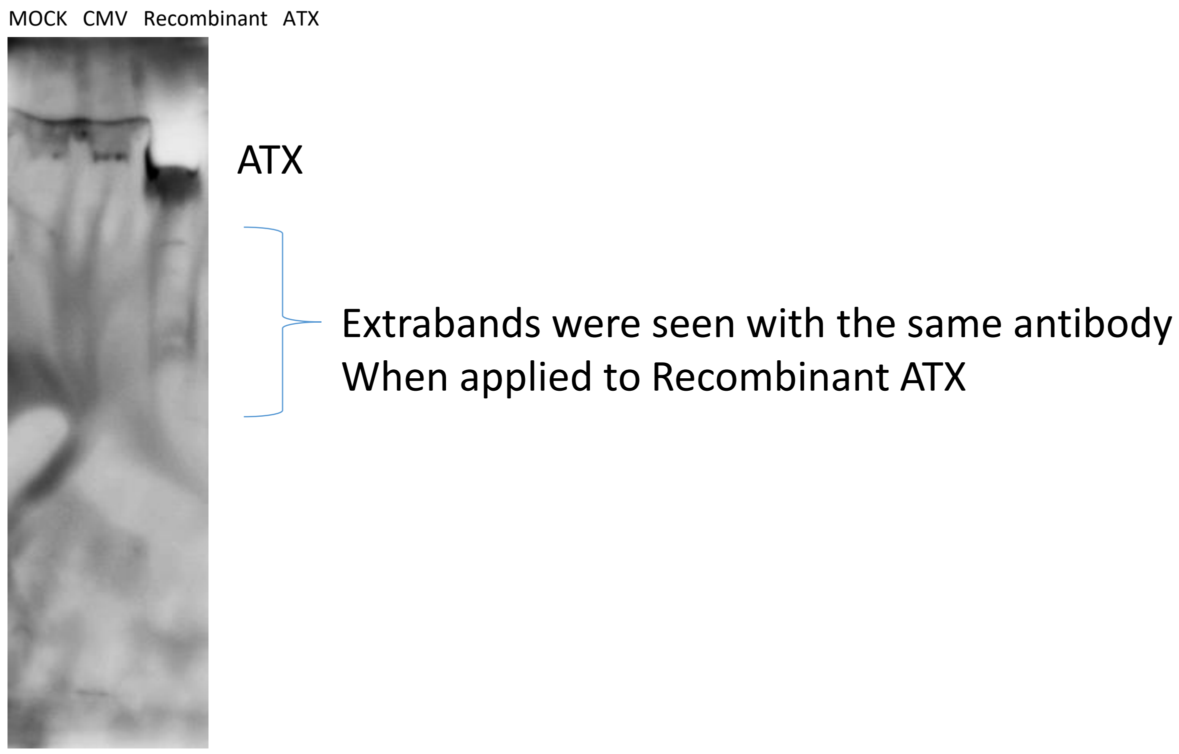

Supplement: Supplementary file 1 — Supplementary Information. [file 41598_2020_63284_MOESM1_ESM.pdf]
